# Serum SH2B1 and SULT1A2 in relation to adiposity and early weight-loss response: a hypothesis-generating study

**DOI:** 10.3389/fendo.2026.1873732

**Published:** 2026-08-06

**Authors:** Haiyan Lv, Guifeng Shi, Yafei Ye, Zhenyu Yang, Jingyun Yang, Meixian Zhang

**Affiliations:** 1 Department of Clinical Laboratory, Taizhou Hospital of Zhejiang Province Affiliated to Wenzhou Medical University, Linhai, China; 2 Department of Public Health, Taizhou Hospital of Zhejiang Province Affiliated to Wenzhou Medical University, Linhai, Zhejiang, China; 3 Health Management Centre, Taizhou Hospital of Zhejiang Province Affiliated to Wenzhou Medical University, Linhai, China; 4 Public Scientific Research Platform, Taizhou Hospital of Zhejiang Province Affiliated to Wenzhou Medical University, Linhai, China; 5 Department of Pediatrics, Taizhou Hospital of Zhejiang Province Affiliated to Wenzhou Medical University, Linhai, China; 6 Evidence-Based Medicine Center, Taizhou Hospital of Zhejiang Province Affiliated to Wenzhou Medical University, Linhai, China

**Keywords:** obesity, weight loss, response, SH2B1, SULT1A2

## Abstract

**Background:**

Individual responses to weight loss interventions are highly heterogeneous. This study aimed to explore the association between serum SULT1A2 and SH2B1 levels and weight loss responses, with the goal of generating biologically relevant hypotheses.

**Methods:**

The first cohort consisted of 223 patients (102 normal weight, 52 overweight, and 69 obese), to assess associations of baselines SULT1A2 and SH2B1 with weight status. The second cohort included 49 eligible participants that completed a 4-week intermittent fasting-based weight management program. They were divided into greater weight loss group (weight loss ≥5% of initial body weight) and lesser weight loss group (weight loss <5%). Serum SULT1A2 and SH2B1 levels were detected by enzyme-linked immunosorbent assay (ELISA).

**Results:**

In the first cohort, serum SULT1A2 and SH2B1 levels varied significantly across BMI groups (*P* = 0.002 and *P* = 0.013, respectively). Both biomarkers were elevated in the overweight group compared with the normal weight group (*P* < 0.01 and *P* < 0.05). The obese group had lower SULT1A2 and SH2B1 levels than the overweight group (*P* < 0.05), with no significant differences versus the normal weight group. In the second cohort, SULT1A2 exhibited no association with weight loss response across both sexes. For SH2B1, male greater weight loss group exhibited significantly higher levels than lesser weight loss group (2477.1 ± 1332.2 vs. 1298.4 ± 649.6 ng/mL, *P* = 0.030, Hedges’ g = 0.97, 95% CI: 0.01–1.97); however, this association was attenuated and did not persist after multivariable adjustment for age and baseline BMI. In females, no such difference was observed (1325.0 ± 812.3 vs. 1135.7 ± 488.3 ng/mL, *P* = 0.471, Hedges’ g = 0.25, 95% CI: −0.49–0.99).

**Conclusions:**

Circulating SULT1A2 and SH2B1 vary across adiposity phenotypes, suggesting their potential involvement in energy metabolism. Baseline SH2B1 showed a male-specific exploratory association with early weight-loss response, although this finding was not independent of age and BMI. Collectively, these results support SH2B1 as a candidate peripheral marker of further investigation in larger, independent cohorts.

## Graphical Abstract

**Figure:**
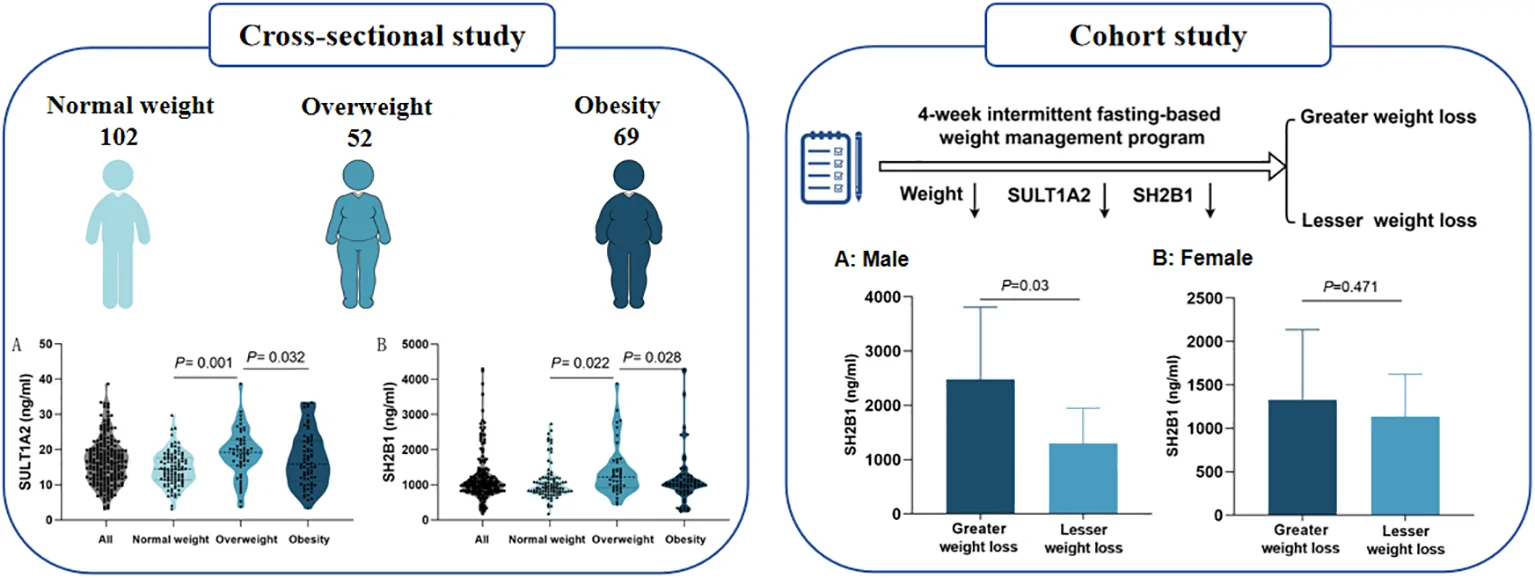

## 1 Introduction

The global prevalence of overweight and obesity has risen continuously over recent decades. In 2022, approximately 43% of adults aged 18 years and older were overweight, and 16% were living with obesity worldwide (1). The Global Burden of Disease Group reported in 2017 that “the prevalence of obesity has doubled in more than 70 countries since 1980 and continues to grow in most others” (2). In China, the proportion of adults who are overweight or obese is projected to reach 63.5% by 2030 (3). Beyond its direct impact on body weight, obesity is frequently accompanied by dyslipidemia and substantially increases the risk of metabolic and cardiovascular complications. Over the next decade, obesity is expected to overtake smoking as a leading risk factor for cancer (4). Moreover, obesity is strongly associated with insulin resistance, type 2 diabetes, non-alcoholic fatty liver disease, and sleep-related breathing disorders such as obstructive sleep apnea (5, 6). Consequently, the prevention and management of obesity have become top priorities in global public health.

National clinical guidelines recommend a weight loss target of at least 10% of initial body weight; notably, even a 5% reduction is associated with substantial and clinically meaningful health benefits (7). Nevertheless, individual responses to weight loss interventions vary widely, and findings from many clinical trials lack generalizability to real-world settings (8). Furthermore, most individuals who achieve weight loss objectives fail to maintain their weight reduction in the long term (9). This heterogeneity and poor sustainability are largely attributable to the complex etiology of obesity, which involves genetic predisposition, epigenetic modifications, and gene-environment interactions (10).

Genetic factors are estimated to contribute up to 70% of obesity susceptibility, and multiple key obesity-related genes have been identified (10). These genes regulate core processes including food intake, dietary preference, energy expenditure, and leptin sensitivity, all of which influence obesity risk and the potential for weight loss (11). For example, the *Ucp1* gene contributes to thermogenesis and regulation of energy expenditure and prevents oxidative stress (12). Despite extensive research into the pathogenesis of obesity and energy homeostasis, current understanding remains insufficient to curb the ongoing global obesity epidemic (13).

The human sulfotransferase family 1A member 2 (*SULT1A2*) gene is located at 16p12.1, which is very close to the obesity research hotspot 16p11.2, and is primarily expressed in the gut and adipose tissue (14). Our recent study demonstrated that the SULT1A2 rs1059491 variant is associated with a lower risk of obesity and dyslipidemia in southern Chinese adults (15), suggesting a potential role for this gene in adiposity-related metabolic traits.

The human SH2B adaptor protein 1 (*SH2B1*, also known as SH2-B and PSM) gene is located at 16p11.2 and plays a pivotal role in energy homeostasis by regulating LepRb/JAK2 signaling (16). SH2B1 is widely expressed in both peripheral tissues and the central nervous system, including adipose tissue, skeletal muscle, liver, hypothalamus, and other brain regions (17). Leptin suppress appetite by concurrently acting on pro-opiomelanocortin (POMC) neurons and neuropeptide Y/agouti-related peptide (NPY/AgRP) neurons, and SH2B1 enhances this leptin-mediated effect (18). Furthermore, SH2B1 may amplify leptin’s appetite-suppressing action by potentiating the ability of brain-derived neurotrophic factor (BDNF) to inhibit appetite-promoting neurons (18). SH2B1 has also been linked to hereditary severe insulin resistance syndrome (19), further underscoring its relevance to metabolic regulation.

Given the established roles of SULT1A2 and SH2B1 in adiposity-related pathways and the limited evidence regarding their circulating levels in relation to weight loss outcomes, we designed the present study as an exploratory investigation to examine whether baseline serum concentrations of these two proteins are associated with adiposity status and early responses to dietary intervention in a Chinese cohort. Rather than testing a predefined clinical predictive model, this study was designed to generate biologically grounded hypotheses that may guide future mechanistic exploration and translational research.

## 2 Materials and methods

### 2.1 Study population

Two cohorts were enrolled in this study. The first cohort comprised 223 participants (102 normal-weight, 52 overweight, and 69 obese individuals) who underwent routine physical examination at the Health Management Center between January 1 and March 10, 2020. All participants had complete anthropometric data and available venous blood samples. Individuals were excluded if they were pregnant or lactating, taking medications known to affect body weight or energy metabolism, or had mental disorders, binge eating disorder, anorexia nervosa, gastrointestinal diseases, or secondary pathological obesity.

**Figure 1:**
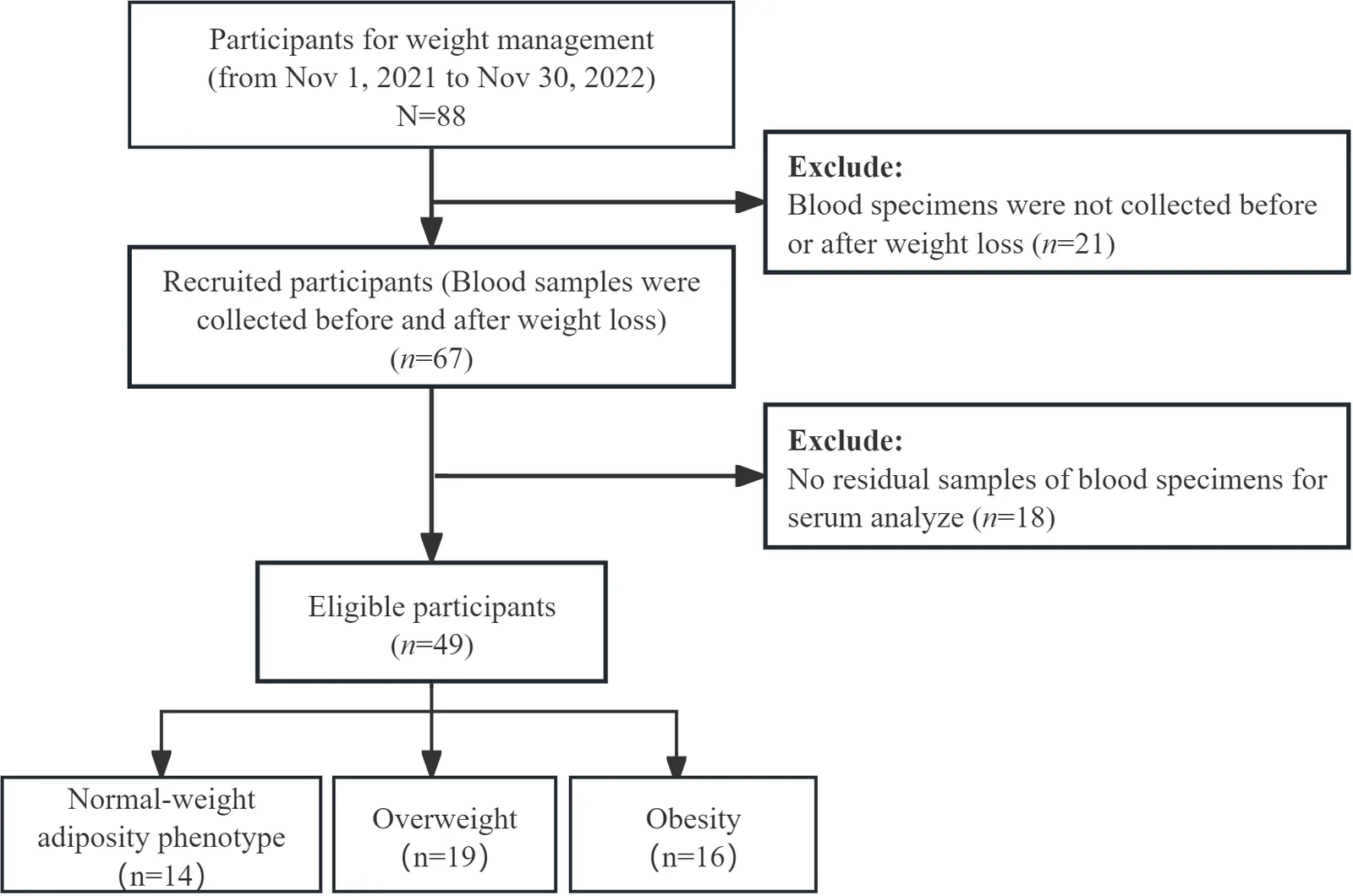
Study design and participant flow diagram (intervention cohort only).

The second cohort included 49 eligible participants (14 normal-weight adiposity phenotype, 19 overweight, and 16 obese individuals) who received standardized weight management between November 1, 2021 and November 30, 2022. Notably, the 14 normal-weight adiposity phenotype subjects qualified for inclusion exclusively due to raised body fat percentage or enlarged waist circumference, consistent with a normal-weight high-adiposity phenotype rather than conventional healthy normal weight. The inclusion criteria were as follows: (1) aged 18–60 years; (2) stable body weight (± 4 kg) in the preceding three months; and (3) meeting at least one of the following criteria: Body mass index (BMI) ≥ 24 kg/m², body fat percentage ≥ 30% (women) or ≥ 25% (men), or waist circumference ≥ 85 cm (women) or ≥ 90 cm (men). The exclusion criteria were as follows: (1) missing pre- or post-intervention blood samples (n = 21); and (2) insufficient residual serum for laboratory analysis (n = 18) (**Figure 1**).

This study was approved by the Medical Ethics Committee of Taizhou Hospital, Zhejiang Province, China (K20210607), and written informed consent was obtained from all enrolled participants.

### 2.2 Weight management program

All participants underwent a 4-week comprehensive weight management program consisting of dietary intervention, exercise training, and cognitive behavioral support, in addition to routine health guidance. Following the 4-week intervention, participants were classified as greater weight loss group if they achieved ≥5% reduction in initial body weight, and lesser weight loss group if weight loss was <5% (20).

#### 2.2.1 Dietary intervention

A modified alternate-day intermittent fasting protocol with nutritional meal replacement was implemented. Participants alternated between fasting days and normocaloric days across the 4-week intervention. On fasting days, only prescribed meal replacement products (whey protein powder and dietary fiber powder; product standard code: GB/T 29602) provided by study dietitians were permitted, with no other food intake allowed except water. On normocaloric days, participants followed a balanced diet under dietitian supervision. Daily total energy intake was restricted to 1200 kcal, and daily water intake was maintained at 1.5–1.8 L, in line with the *2022 Dietary Guidelines for Chinese Residents*.

#### 2.2.2 Exercise intervention

Participants were required to complete at least 10,000 steps per day.

#### 2.2.3 Cognitive behavioral and psychological support

A multidisciplinary “three-in-one management” model was implemented, involving endocrinologists, dietitians, and health managers. Participants maintained daily records of body weight, dietary intake, and physical activity, and reported these data daily in a dedicated WeChat group. Regular nutrition and health education was provided to address physiological and psychological challenges during weight management.

### 2.3 Safety evaluation

Safety was assessed by monitoring adverse events throughout the intervention. Adverse event questionnaires were completed weekly, and safety was evaluated using the incidence of adverse events (calculated as: number of participants with adverse events/total number of participants×100%). Potential adverse events were defined as follows: (1) Hunger: Hunger, fatigue, palpitations, or other discomfort related to insufficient energy intake. (2) Hypoglycemia: Documented hypoglycemic events, including dizziness, pale complexion, cold sweats, limb weakness, hypotension, nausea, vomiting, or blood glucose < 2.8 mmol/L.(3) Gastrointestinal discomfort: Constipation, diarrhea, dry mouth, abdominal bloating, or other gastrointestinal symptoms related to dietary changes. No hypoglycemia, excessive hunger, gastrointestinal distress, or other metabolic adverse events were reported during the entire intervention period.

### 2.4 Compliance evaluation

Compliance was evaluated based on adherence to the intermittent fasting protocol and moderate exercise program. After consultation with a study dietitian, all participants provided written informed consent and agreed to strictly comply with the weight management program. Participants were required to upload daily records of dietary intake, physical activity, and body weight to a dedicated WeChat group at scheduled time points. Regular follow-up was performed throughout the intervention.

Compliance was verified by counting unused meal replacement products and reviewing daily submitted diet and activity logs. Attrition was used to assess overall adherence, with the dropout rate calculated as follows (dropout rate = number of participants lost to follow-up at the final visit/total number of participants included in the analysis × 100%). Among the 88 initially enrolled participants, 21 dropped out during the study, resulting in a 23.86% attrition rate (**Figure 1**).

### 2.5 Measurements

As described in detail previously[15], anthropometric data were collected by trained healthcare technicians using standardized protocols. Body weight was measured to the nearest 0.1 kg on a balance beam scale in light clothing, standing unshod at the first visit, and every two weeks for four weeks. Height was measured to the nearest 0.1 cm with a wall-mounted stadiometre. BMI was calculated as the weight in kilograms divided by the square of height in meters. According to the Chinese criteria, BMI ≥ 28 kg/m^2^ was considered obese, and 24 kg/m^2^ ≤ BMI < 28 kg/m^2^ was considered overweight for adults.

An electronic blood pressure monitor (Omron, HBP-9021) was used to measure the blood pressure in the right upper arm. Blood pressure values with systolic blood pressure (SBP) ≥ 140 mmHg, diastolic blood pressure (DBP) ≥ 90 mmHg, or reported use of antihypertensive medication could indicate hypertension. We used the InBody770 bioimpedance analyzer (Seoul, Korea) (21) to determine body composition indices including body fat percentage, body fat mass, and visceral fat area. All subjects maintained a standard posture by standing on metal footplates and holding the instrument handles throughout measurements. All body composition measurements before and after the weight loss intervention were performed in the morning under fasting conditions.

### 2.6 Blood sample collection and biochemical analysis

Approximately 3 ml of peripheral venous blood was collected in the morning from subjects who had fasted for 12 hours. Serum samples were collected for biochemical testing after non-anticoagulant centrifugation. The hexokinase method was used to measure fasting plasma glucose (FPG). Enzyme-coupled calorimetry was used to detect the serum total cholesterol (TC), high-density lipoprotein cholesterol (HDL-C), low-density lipoprotein cholesterol (LDL-C), and triglyceride (TG) levels. Dyslipidemia was diagnosed by the presence of one or more of the following components: TC ≥ 5.20 mmol/L (200 mg/dL), LDL-C ≥ 3.40 mmol/L (130 mg/dL), HDL-C < 1.00 mmol/L (40 mg/dL), TG ≥ 1.70 mmol/L (150 mg/dL), or the use of anti-dyslipidemia medication in accordance with the 2016 Chinese Adult Dyslipidemia Management Guidelines (22). Impaired fasting glucose (IFG) was defined as an FPG ≥ 5.60 mmol/L.

### 2.7 ELISA quantification of SH2B1 and SULT1A2

Serum levels of SH2B1 and SULT1A2 were measured by enzyme-linked immunosorbent assay (ELISA) using the monoclonal antibodies SH2B1 and SULT1A2 ELISA Kit (Abbexa, Catalogue No: abx383165 and abx547527), respectively, according to the manufacturer’s instructions. The commercially available ELISA kits for human SULT1A2 and SH2B1 were officially validated for serum testing by their respective manufacturers. Both reagents target all isoforms and share an identical detection range of 0.156-10 ng/mL, with lower limits of detection of 0.08 ng/mL (SULT1A2) and 0.094 ng/mL (SH2B1). Prior to measurement, serum specimens were diluted at 1:20 for SULT1A2 testing and 1:1000 for SH2B1 testing, and all samples were assayed in duplicate to ensure reliable results.

In summary, 100 µL aliquots of the diluted samples were added to the sample wells and incubated for 1 h at 37 °C. The samples were washed three times with wash buffer after incubation with detection reagent A for 1h at 37°C. Detection reagent B (100 µL) was aliquoted into each well, and the samples were incubated for 90 min at 37 °C. After washing five times with wash buffer, 90 µL of TMB substrate was added to each well and the ELISA plate was incubated in the dark for 10-20 min at 37 °C. Then, 50 µL of stop solution was added and the absorbance of each sample was measured at 450 nm.

Intra-assay precision (Precision within an assay) was evaluated by testing low, medium, and high levels of SULT1A2 and SH2B1 in 3 samples with 20 repeated measurements on the same plate. Besides, Inter-assay precision (Precision between assays) was assessed using the same 3 samples analyzed on 3 separate plates, with 8 replicates per plate. The coefficient of variation (CV) was calculated as: CV (%) = (Standard Deviation/Mean)×100. Intra-assay CV was < 7% and inter-assay CV was < 10% for both markers.

### 2.8 Statistical analysis

The normality of all continuous variables was assessed using the Shapiro–Wilk test. Most variables were approximately normally distributed across weight categories (*P* > 0.05 for all groups), with the exception of TG, SULT1A2, and SH2B1, which showed significant deviations from normality. Therefore, data with non-normal distribution were described as the geometric mean (interquartile range, IQR), and logarithmic transformation was applied to these three variables prior to group comparisons.

Continuous data are presented as adjusted mean ± standard deviation (SD) unless otherwise specified. Comparisons of continuous variables across the three weight categories (normal-weight adiposity phenotype, overweight, and obese) were performed using analysis of covariance (ANCOVA), with age and sex included as covariates. Following a significant omnibus F-test, post-hoc pairwise comparisons were conducted using Bonferroni correction test. All three pairwise comparisons (normal-weight adiposity phenotype vs. overweight, normal-weight adiposity phenotype vs. obese, and overweight vs. obese) are reported, including non-significant pairs. Categorical variables regarding basic demographic characteristics were expressed as counts and percentages and were compared among groups using chi-square tests.

Paired *t*-tests were used to detect differences in anthropometric indices, cardiometabolic parameters, and SULT1A2 and SH2B1 levels before and after weight loss. A *t*-test was performed to compare baseline anthropometric and cardiometabolic parameters between the high and low responders. Spearman correlation coefficient were used to assess correlations between baseline SH2B1 levels and weight loss outcomes. Multivariable logistic regression adjusted for age and baseline BMI was performed to identify the association between SH2B1 and weight loss response.

To assess the robustness of our findings, two sensitivity analyses were conducted. Initially, we applied the Mann–Whitney U test for non-parametric comparisons of baseline biomarkers between high and low responders within each sex group. Subsequently, we excluded 14 females with normal BMI but elevated waist circumference or body fat percentage, and then repeated all sex-stratified analyses in the subsample restricted to individuals with BMI-defined overweight or obesity.

All the data were analyzed using IBM SPSS version 29.0 and the differences were considered statistically significant at *P* < 0.05.

## 3 Results

### 3.1 Serum SULT1A2 and SH2B1 levels across adiposity phenotypes in the first cohort

Baseline clinical characteristics of participants in the first cohort are summarized in **Table 1**. Apart from age, height, and fasting plasma glucose (FPG), significant intergroup differences were observed in multiple obesity-associated parameters, including blood pressure (*P* < 0.001), triglyceride (TG, *P* < 0.001), high-density lipoprotein cholesterol (HDL-C, *P* < 0.001), and low-density lipoprotein cholesterol (LDL-C, *P* < 0.05).

**Table 1:** Characteristics of the study population at baseline (*n*=223).

Variables	All	Normal weight	Overweight	Obesity	*P*
	(*n*=223)	(*n*=102, 45.74%)	(*n*=52, 23.32%)	(*n*=69, 30.94%)	
Male [n (%)]	90 (40.4)	27 (26.5)	16 (30.8)	47 (68.1)	**<0.001**
Age (years)	37.5 ± 10.9	36.3 ± 9.5	38.2 ± 10.7	38.7 ± 12.8	0.341
Height (cm)	164.2 ± 8.5	162.7 ± 7.5	163.2 ± 8.8	167.3 ± 8.9	0.806
Weight (kg)	72.5 ± 17.5	58.6 ± 7.4	74.2 ± 9.2***	91.9 ± 13.7***^△△△^	**<0.001**
BMI (kg/m^2^)	26.7 ± 5.0	22.1 ± 1.8	27.8 ± 1.4***	32.7 ± 2.5***^△△△^	**<0.001**
SBP (mmHg)	125 ± 16	118 ± 13	130 ± 18***	133 ± 14***	**<0.001**
DBP (mmHg)	75 ± 13	69 ± 9	78 ± 14***	81 ± 13***	**<0.001**
TG (mmol/L)	1.40 (0.89−2.03)	1.01 (0.78−1.34)	1.63 (1.01−2.44)***	2.02 (1.42−3.13)***	**<0.001**
TC (mmol/L)	4.87 ± 0.92	4.65 ± 0.93	5.10 ± 0.93*	5.00 ± 0.82	**0.02**
HDL-C (mmol/L)	1.36 ± 0.32	1.51 ± 0.33	1.30 ± 0.24***	1.18 ± 0.26***	**<0.001**
LDL-C (mmol/L)	2.57 ± 0.68	2.38 ± 0.66	2.74 ± 0.75*	2.68 ± 0.59	**0.011**
FPG (mmol/L)	5.46 ± 1.60	5.20 ± 0.55	5.50 ± 1.97	5.81 ± 2.15	0.335
SULT1A2 (ng/mL)	15.03 (11.74−19.87)	13.70 (11.34−17.67)	17.58 (15.57−22.74)**	15.30 (11.04−22.38)△	**0.002**
SH2B1 (ng/mL)	1035.6 (829.0−1244.9)	978.0 (804.8−1176.8)	1246.1 (918.4−1630.7)*	996.9 (833.0−1193.8)^△^	**0.013**

Data are presented as mean ± SD for untransformed variables, or as geometric mean (interquartile range, IQR) for log-transformed variables (SULT1A2, SH2B1 and TG), after adjustment for age and sex. Obesity was defined using the International Obesity Task Force (IOTF) BMI cutoffs. P values were derived from ANCOVA with age and sex as covariates. Post-hoc comparisons:*P < 0.05, **P < 0.01, ***P < 0.001 vs. normal weight group; △P < 0.05, △△△P < 0.001 vs. overweight group.

Bold values represent statistically significant results with *P* < 0.05.

**Figure 2:**
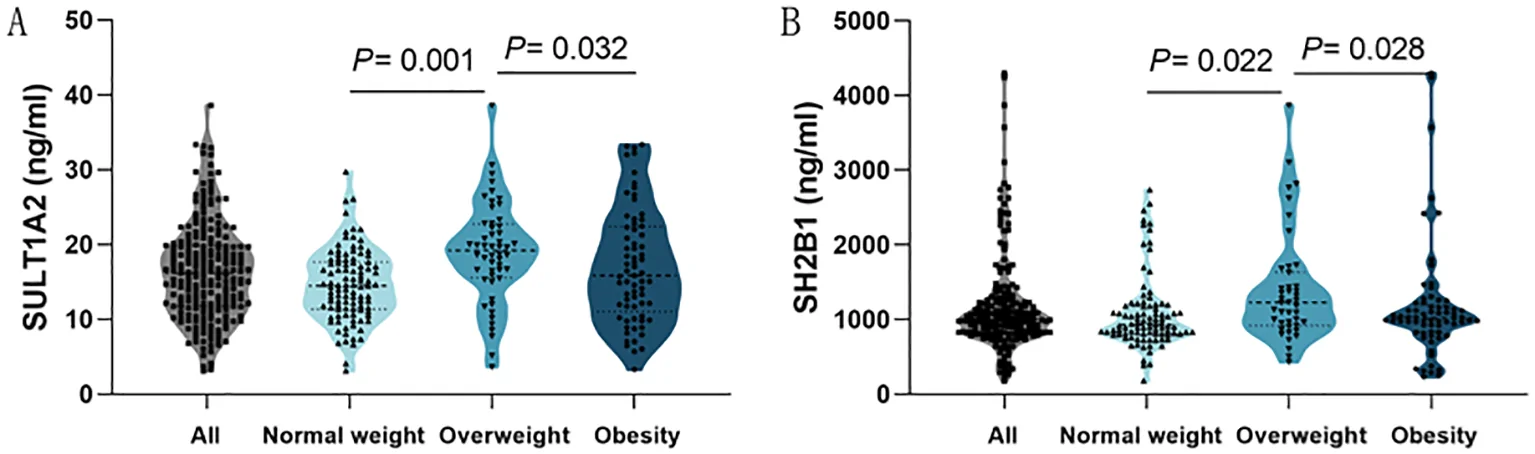
Comparison of serum SULT1A2 and SH2B1 levels among participants with normal weight, overweight and obesity. **(A)** SULT1A2. **(B)** SH2B1.

The overall median serum SULT1A2 level across all participants was 15.03 ng/mL (IQR: 11.74–19.87 ng/mL). Serum SULT1A2 levels differed significantly across the normal-weight, overweight, and obese groups (*P* = 0.002). *Post-hoc* analyses revealed that the overweight group had markedly higher SULT1A2 levels [17.58 (15.57–22.74) ng/mL] than the normal-weight group [13.70 (11.34–17.67) ng/mL, *P* < 0.01]. No significant difference was observed between the obese group and the normal-weight group, whereas the obese group showed lower SULT1A2 levels compared with the overweight group (*P* < 0.05), as presented in **Table 1** and **Figure 2A**.

For serum SH2B1, the overall median level was 1035.6 ng/mL (IQR: 829.0–1244.9 ng/mL), with a significant difference among the three groups (*P* = 0.013). *Post-hoc* comparisons showed that the overweight group had significantly higher SH2B1 levels [1246.1 (918.4–1630.7) ng/mL] than the normal-weight group [978.0 (804.8–1176.8) ng/mL, *P* < 0.05]. There was no significant difference in SH2B1 levels between the obese group and the normal-weight group. Meanwhile, the obese group [996.9 (833.0–1193.8) ng/mL] exhibited lower SH2B1 concentrations relative to the overweight group (*P* < 0.05), as shown in **Table 1** and **Figure 2B**.

### 3.2 Baseline characteristics of participants in the second cohort

A total of 49 participants completed the 4-week weight management intervention, of whom, 17 (34.7%) were male. Significant differences in sex distribution were observed across the normal-weight, overweight, and obese groups (*p* < 0.002; **Table 2**). Prior to the intervention, no remarkable intergroup differences were observed regarding age, hypertension, impaired fasting glucose (IFG), elevated TG, elevated total cholesterol (TC), elevated LDL-C, reduced HDL-C, and dyslipidemia among the three groups (**Table 2**).

**Table 2:** Baseline characteristics of participants in weight management (*n*=49).

Variables	All (*n*=49)	Normal- weight adiposity phenotype (*n*=14)	Overweight (*n*=19)	Obesity (*n*=16)	*P*
Male [n (%)]	17 (34.7)	0	7 (36.8)	10 (62.5)	**<0.002**
Age (years)	36.5 ± 10.9	37.0 ± 10.9	34.8 ± 10.1	37.9 ± 12.2	0.696
High SBP (SBP≥140 mmHg)	9 (18.4)	0	5 (26.3)	4 (25.0)	0.091
High DBP (DBP≥90 mmHg)	9 (18.4)	0	5 (26.3)	4 (25.0)	0.091
IFG (FPG≥5.60 mmol/L)	10 (20.4)	2 (14.3)	3 (15.8)	5 (31.3)	0.491
High TG (fasting triglycerides≥1.7 mmol/L)	12 (24.5)	2 (14.3)	4 (21.1)	6 (37.5)	0.377
High TC (fasting TC≥5.2 mmol/L)	18 (36.7)	4 (28.6)	7 (36.8)	7 (43.8)	0.653
High LDL-C (fasting LDL-C≥3.40 mmol/L)	4 (8.2)	0	2 (10.5)	2 (12.5)	0.538
Low HDL-C (HDL-C<1.0 mmol/L)	4 (8.2)	1 (7.1)	1 (5.3)	2 (12.5)	0.819
Dyslipidaemia	24 (49.0)	5 (35.7)	10 (52.6)	9 (56.3)	0.552

SBP, systolic blood pressure; DBP, diastolic blood pressure; FPG, fasting plasma glucose; TC, total cholesterol; HDL-C, high-density lipoprotein cholesterol; LDL-C, low-density lipoprotein cholesterol.

Dyslipidaemia was defned by the presence of one or more of the following components conditions: TC≥5.20 mmol/L (200 mg/dL), LDL-C≥3.40 mmol/L (130 mg/dL), HDL-C<1.00 mmol/L (40 mg/dL), TG≥1.70 mmol/L (150 mg/dL), or if they were taking anti-dyslipidaemia medication.

Bold values represent statistically significant results with *P* < 0.05.

### 3.3 Changes in adiposity parameters, cardiometabolic indices, and serum SULT1A2 and SH2B1 levels after the 4-week intervention

Following 4 weeks of weight management, significant reductions were observed in body weight (71.5 ± 13.1 vs. 75.7 ± 13.9 kg), BMI (26.3 ± 3.7 vs. 27.9 ± 3.9 kg/m²), abdominal circumference (90.9 ± 9.4 vs. 96.5 ± 10.3 cm), and visceral fat area (111.7 ± 34.4 vs. 133.5 ± 36.2 cm²) compared with baseline (all *P* < 0.001; **Table 3**). Meanwhile, serum FPG (4.95 ± 0.88 vs. 5.30 ± 1.16 mmol/L, *P* = 0.004), TG (1.20 ± 0.88 vs. 1.95 ± 2.06 mmol/L, *P* = 0.004), TC (4.47 ± 0.93 vs. 4.87 ± 0.92 mmol/L, *P* < 0.001), and LDL-C (2.29 ± 0.61 vs. 2.46 ± 0.64 mmol/L, *P* = 0.004) were markedly decreased compared with the baseline levels. Overall, most adiposity and cardiometabolic parameters improved notably, whereas HDL-C showed no significant change. Furthermore, significant reductions in serum SULT1A2 (16.48 ± 7.46 vs. 18.56 ± 6.20 ng/mL, *P* = 0.018) and SH2B1 (1123.9 ± 411.1 vs. 1515.2 ± 985.3 ng/mL, *P* = 0.010) concentrations were observed after weight management compared with baseline (**Table 3**).

**Table 3:** Baseline values and changes in adiposity parameters and cardiometabolic indices (mean ± SD) after 4 week diet intervention (*n*=49).

Variables	Baseline	After 4 weeks	Reduce from baseline	*P*
Weight (kg)	75.7 ± 13.9	71.5 ± 13.1	4.2 ± 2.1	**<0.001**
BMI (kg/m2)	27.9 ± 3.9	26.3 ± 3.7	1.5 ± 0.7	**<0.001**
Percent Body Fat (%)	36.4 ± 6.1	33.5 ± 6.5	2.9 ± 1.5	**<0.001**
Abdomen Circumference (cm)	96.5 ± 10.3	90.9 ± 9.4	5.6 ± 2.5	**<0.001**
Waist Hip Ratio	0.95 ± 0.05	0.92 ± 0.05	0.04 ± 0.02	**<0.001**
Visceral Fat Area (cm^2^)	133.5 ± 36.2	111.7 ± 34.4	21.9 ± 8.5	**<0.001**
SBP (mmHg)	128 ± 20	120 ± 18	8 ± 13	**<0.001**
DBP (mmHg)	77 ± 17	74 ± 13	3 ± 10	**0.038**
Pulse (beat/min)	80 ± 10	74 ± 10	7 ± 9	**<0.001**
FPG (mmol/L)	5.30 ± 1.16	4.95 ± 0.88	0.35 ± 0.81	**0.004**
TG (mmol/L)	1.95 ± 2.06	1.20 ± 0.88	0.75 ± 1.72	**0.004**
TC (mmol/L)	4.87 ± 0.92	4.47 ± 0.93	0.40 ± 0.62	**<0.001**
LDL-C (mmol/L)	2.46 ± 0.64	2.29 ± 0.61	0.17 ± 0.4	**0.004**
HDL-C (mmol/L)	1.32 ± 0.27	1.31 ± 0.25	0.01 ± 0.16	0.578
SULT1A2 (ng/mL)	18.56 ± 6.20	16.48 ± 7.46	2.08 ± 5.96	**0.018**
SH2B1 (ng/mL)	1515.2 ± 985.3	1123.9 ± 411.1	391.2 ± 1008.3	**0.010**

BMI, body mass index; SBP, systolic blood pressure; DBP, diastolic blood pressure; FPG, fasting plasma glucose; TC, total cholesterol; HDL-C, high-density lipoprotein cholesterol; LDL-C, low-density lipoprotein cholesterol.

Bold values represent statistically significant results with *P* < 0.05.

### 3.4 Baseline characteristics and serum SULT1A2 and SH2B1 levels stratified by weight loss response

A total of 32 female participants were enrolled, with 20 (62.5%) achieving ≥5% weight loss (greater weight loss group) and 12 (37.5%) achieving <5% weight loss (lesser weight loss group) (**Table 4**). Baseline serum SULT1A2 levels showed no significant difference between the two groups (15.77 ± 5.95 ng/mL vs. 16.39 ± 3.56 ng/mL, *P* = 0.748). Similarly, baseline SH2B1 concentrations did not differ significantly in females (1325.0 ± 812.3 ng/mL vs. 1135.7 ± 488.3 ng/mL, *P* = 0.471). Of note, female participants in the greater weight loss group had significantly higher baseline TG levels than those in the lesser weight loss group (1.82 ± 1.36 mmol/L vs. 1.20 ± 0.67 mmol/L, *P* = 0.024). No other baseline indicators differed significantly between female subgroups.

**Table 4:** Baseline anthropometric and cardiometabolic parameters for female in the greater and lesser weight loss response to the diet intervention (n=32).

Variables	Greater weight loss group	Lesser weight loss group	*MD* (High−Low)	*MD* 95%*CI*	*P*	*Cohen's d*	*Hedges’g*	*Hedges' g* 95%*CI*
	*n*=20	*n*=12						
Body weight (kg)	70.2±8.5	66.0±6.2	4.20	(-0.92–9.32)	0.149	0.54	0.53	-0.22–1.28
BMI (kg/m2)	27.5±3.3	25.4±2.7	2.10	(-0.004–4.204)	0.081	0.68	0.66	-0.08–1.42
SBP (mmHg)	123.2±16.4	120.9±17.8	2.30	(-10.073–14.673)	0.696	0.14	0.13	-0.61–0.87
DBP (mmHg)	73.4±12.8	70.8±14.8	2.60	(-7.479–12.679)	0.616	0.192	0.18	-0.56–0.92
Pulse (beat/min)	78.0±8.6	78.9±6.2	-0.90	(-6.049–4.249)	0.849	-0.11	-0.11	-0.85–0.63
FPG (mmol/L)	5.12±1.01	5.10±0.49	0.02	(-0.502–0.542)	0.63	0.02	0.02	-0.72–0.76
TG (mmol/L)	1.82±1.36	1.20±0.67	0.62	(-0.086–1.326)	0.024	0.54	0.53	-0.22–1.28
TC (mmol/L)	4.75±0.92	4.83±1.05	-0.08	(-0.798–0.638)	0.891	-0.08	-0.08	-0.82–0.66
LDL-C (mmol/L)	2.41±0.61	2.45±0.74	-0.04	(-0.537–0.457)	0.779	-0.06	-0.06	-0.80–0.68
HDL-C (mmol/L)	1.34±0.20	1.50±0.32	-0.16	(-0.361–0.041)	0.226	-0.64	-0.62	-1.39–0.13
Body Fat Mass	27.8±6.6	24.5±4.4	3.30	(-0.516–7.116)	0.137	0.56	0.55	-0.20–1.30
Percent Body Fat	39.3±5.2	37.0±4.1	2.30	(-0.952–5.552)	0.188	0.48	0.46	-0.28–1.20
Abdomen Circumference	94.2±8.4	90.3±6.4	3.90	(-1.264–9.064)	0.159	0.50	0.49	-0.25–1.23
Waist Hip Ratio	0.95±0.05	0.94±0.04	0.01	(-0.022–0.042)	0.456	0.22	0.21	-0.53–0.95
Visceral Fat Area	141.1±37.7	123.7±28.9	17.40	(-5.846–40.646)	0.163	0.50	0.48	-0.26–1.22
SULT1A2 (ng/mL)	15.77±5.95	16.39±3.56	-0.62	(-3.915–2.675)	0.748	-0.12	-0.12	-0.86–0.62
SH2B1 (ng/mL)	1325±812.3	1135.7±488.3	189.30	(-261.34–639.94)	0.471	0.27	0.25	-0.49–0.99

Participants were classified as greater weight loss group if they achieved ≥5% reduction in initial body weight, and lesser weight loss group if weight loss was <5%.

**Table 5** displayed the data from 17 male participants, among whom 10 (58.8%) were in the greater weight loss group and 7 (41.2%) in the lesser weight loss group. There was no significant difference in baseline SULT1A2 levels between the two male groups (22.42 ± 5.03 ng/mL vs. 24.75 ± 5.37 ng/mL, *P* = 0.375). By contrast, an exploratory observation emerged in males: baseline SH2B1 levels were notably higher in those with greater weight loss compared with those with lesser weight loss (2477.1 ± 1332.2 ng/mL vs. 1298.4 ± 649.6 ng/mL, *P* = 0.030), with a Cohen’s d of 1.06 and a small-sample corrected Hedges’g of 0.97 (95% CI: 0.01–1.97). No significant difference in TG levels was observed between the two male groups (3.16 ± 2.99 mmol/L vs. 2.00 ± 1.43 mmol/L, *P* = 0.336). Likewise, other baseline parameters including blood pressure, FPG, LDL-C, HDL-C and body fat mass were comparable between the two male groups.

**Table 5:** Baseline anthropometric and cardiometabolic parameters for male in the greater and lesser weight loss response to the diet intervention (n=17).

Variables	Greater weight loss group	Lesser weight loss group						
	*n*=10	*n*=7	*MD* (High−Low)	*MD* 95%*CI*	*P*	*Cohen's d*	*Hedges’g*	*Hedges' g* 95%*CI*
Body weight (kg)	89.8±7.7	92.3±13.5	-2.50	-13.58–8.58	0.594	-0.24	-0.20	-1.18–0.78
BMI (kg/m^2^)	30.6±3.1	31.1±3.5	-0.50	-3.73–2.73	0.678	-0.15	-0.14	-1.12–0.84
SBP (mmHg)	136.9±19.5	132.1±22.5	4.80	-15.79–25.39	0.704	0.23	0.21	-0.77–1.19
DBP (mmHg)	85.4±18.7	79.9±15.8	5.50	-10.97–21.97	0.537	0.31	0.28	-0.70–1.26
Pulse (beat/min)	83.3±11.1	83±11.3	0.30	-10.54–11.14	0.825	0.03	0.03	-0.95–1.01
FPG (mmol/L)	5.64±1.45	6.19±3.53	-0.55	-3.32–2.22	0.613	-0.22	-0.18	-1.16–0.80
TG (mmol/L)	3.16±2.99	2.00±1.43	1.16	-0.97–3.29	0.336	0.47	0.46	-0.54–1.46
TC (mmol/L)	4.97±0.86	4.89±0.86	0.08	-0.75–0.91	0.79	0.09	0.09	-0.89–1.07
LDL-C (mmol/L)	2.55±0.70	2.71±0.57	-0.16	-0.77–0.45	0.37	-0.25	-0.23	-1.21–0.75
HDL-C (mmol/L)	1.10±0.16	1.13±0.21	-0.03	-0.21–0.15	0.76	-0.17	-0.15	-1.13–0.83
Body Fat Mass	28.6±6.2	32.5±6.7	-3.90	-10.18–2.38	0.258	-0.61	-0.57	-1.57–0.43
Percent Body Fat	31.6±5.0	34.9±3.6	-3.30	-7.39–0.79	0.182	-0.73	-0.68	-1.68–0.32
Abdomen Circumference	104.0±8.8	106.0±10.7	-2.00	-11.62–7.62	0.841	-0.21	-0.19	-1.17–0.79
Waist Hip Ratio	0.97±0.06	0.97±0.05	0.00	-0.05–0.05	0.974	0	0	-0.98–0.98
Visceral Fat Area	127.9±33.5	146.6±31.8	-18.70	-50.10–12.70	0.333	-0.57	-0.53	-1.53–0.47
SULT1A2 (ng/mL)	22.42±5.03	24.75±5.37	-2.33	-7.38–2.72	0.375	-0.45	-0.41	-1.41–0.59
SH2B1 (ng/mL)	2477.1±1332.2	1298.4±649.6	1178.70	222.99–2134.41	**0.03**	1.06	0.97	0.01–1.97

Participants were classified as greater weight loss group if they achieved ≥5% reduction in initial body weight, and lesser weight loss group if weight loss was <5%.

Bold values represent statistically significant results with *P* < 0.05.

### 3.5 Sensitivity and multivariable-adjusted analyses

Non-parametric testing yielded no significant differences in SULT1A2 and SH2B1 levels between female response groups (n = 32). In males (n = 17), the difference in SH2B1 was of borderline significance (*P* = 0.088) with a wide 95% CI, suggesting limited analytical precision. All median differences and 95% CIs for stratified analyses are summarized in **Supplementary Tables 1**, **2**.

To further evaluate the robustness of these findings, we performed sex-stratified analyses after excluding 14 participants with normal-weight adiposity (**Supplementary Tables 3**, **4**). In females (n=18), no differences were observed in baseline SULT1A2 and SH2B1 between the two response groups (all *P >*0.05). In males (n = 17), the results remained consistent with the primary analysis: no difference in SULT1A2 (*P* = 0.375), and higher SH2B1 levels in the greater weight loss group (*P* = 0.030).

Crucially, correlation analysis showed that baseline SH2B1 levels were not significantly correlated with the actual percentage of weight loss (r = 0.202, *P* > 0.05).

Furthermore, after multivariable adjustment for age and baseline BMI, serum SH2B1 was no longer significantly associated with weight loss response in the overall population, in males, or in females (all *P* > 0.05; **Supplementary Table 5**). Similarly, SULT1A2 showed no significant association with weight loss outcomes in any group, before or after adjustment (all *P* > 0.05).

**Figure 3:**
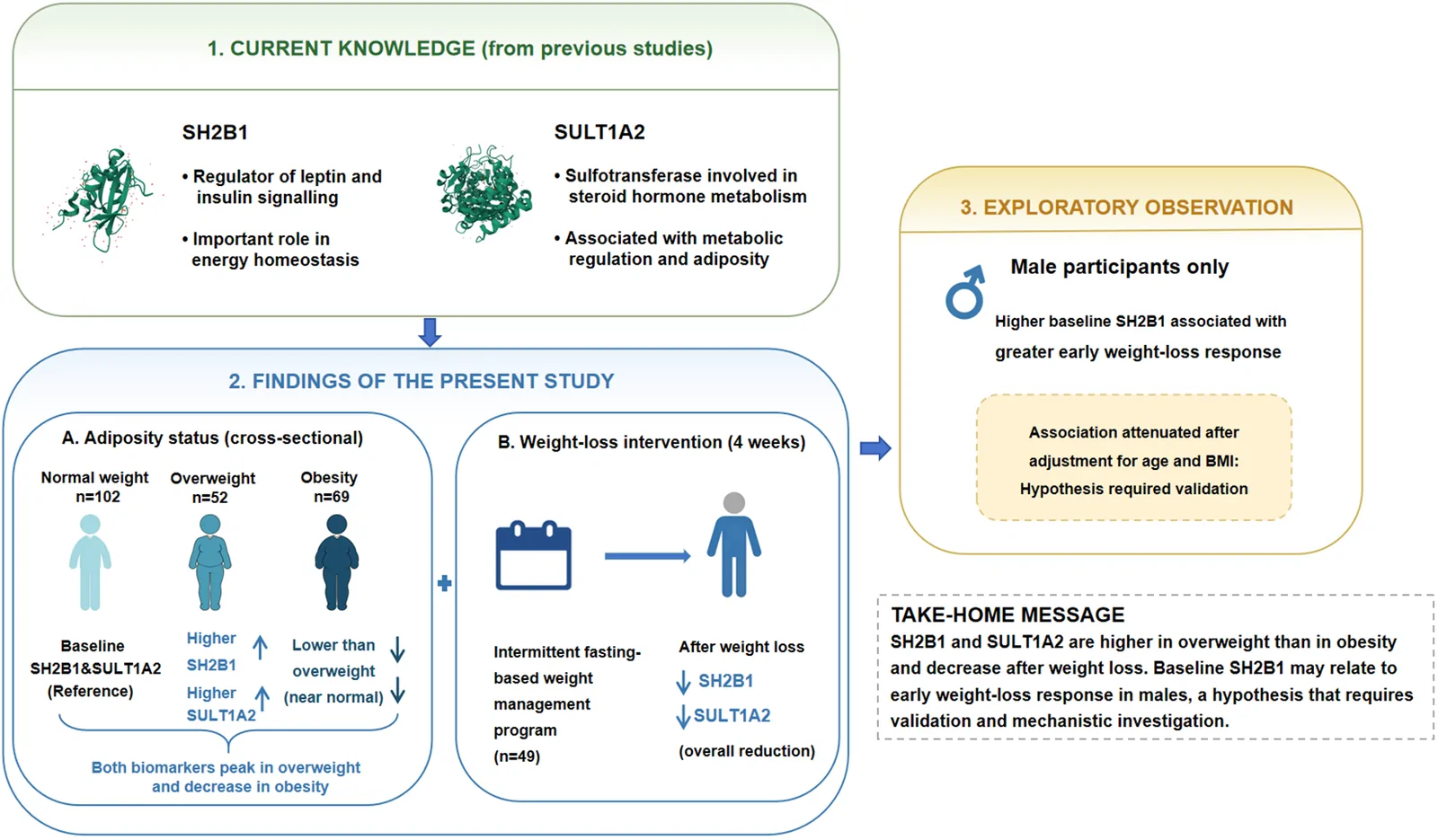
Conceptual schematic summary integrating prior evidence, cross-sectional and intervention-derived exploratory analyses, subgroup observations, and primary interpretative takeaways from this hypothesis-generating study of circulating SH2B1 and SULT1A2 in relation to adiposity and early weight change during intermittent fasting. 1. Current established background from published work: SH2B1 mediates leptin and insulin signaling and participates in systemic energy homeostasis regulation; SULT1A2 is a sulfotransferase enzyme governing steroid hormone turnover, with documented links to metabolic profiles and adiposity. 2. Primary exploratory findings from the present investigation, split into two complementary study arms: **(A)** Cross-sectional adiposity stratification cohort (total n=223: normal weight n=102, overweight n=52, obesity n=69). Circulating SH2B1 and SULT1A2 concentrations display a consistent exploratory pattern: both biomarkers peak among overweight individuals, with reduced concentrations observed in participants with obesity, approximating levels seen in normal-weight controls. **(B)** Four-week intermittent fasting weight management exploratory intervention cohort (n=49). Across all participants, post-intervention reductions in serum SH2B1 and SULT1A2 were observed as a collective exploratory trend. 3. Sex-stratified exploratory subgroup observation limited to male participants only: Unadjusted baseline SH2B1 concentrations appeared higher among male with more pronounced early weight loss; this crude observational association diminished substantially following multivariable adjustment for age and baseline BMI, indicating this preliminary observation requires independent validation rather than representing a definitive predictive marker. Consolidated take-home interpretation: Circulating SH2B1 and SULT1A2 exhibit divergent concentration patterns across distinct adiposity phenotypes and show overall declines following short-term calorie-restricted weight management. A preliminary unadjusted association between baseline SH2B1 and early weight change was only apparent in male participants; this exploratory finding merits further mechanistic and independent cohort investigation without conclusive predictive inference at this stage.

To contextualize our findings within the known metabolic roles of SH2B1 and SULT1A2, we generated a conceptual schematic outlining the cross-sectional biomarker patterns and intervention-induced changes identified in the present work (**Figure 3**).

## 4 Discussion

The present study provides exploratory evidence that circulating levels of SULT1A2 and SH2B1 fluctuate in relation to adiposity status and, in the case of SH2B1, may show a sex-dimorphic association with early weight-loss outcomes. In the first cohort, serum SULT1A2 and SH2B1 levels were significantly elevated in individuals with overweight compared with those of normal weight. Consistent with these findings, both proteins decreased substantially following a 4-week weight-loss intervention in the second cohort. Additionally, baseline serum SH2B1 concentration was associated with the magnitude of weight reduction in males. However, it is crucial to interpret these findings with caution. The association between SH2B1 and weight loss did not persist adjustment for age and BMI in more conservative models, and its robustness was limited in sensitivity analyses. Therefore, rather than validating a predictive biomarker, our results should be viewed as generating biologically testable hypotheses that require independent replication in larger, well-phenotyped cohorts before any clinical inference can be drawn.

### 4.1 Baseline SULT1A2 and obesity: an exploratory observation

The chromosomal locus 16p11.2, which harbors SULT1A2 together with SH2B1 and three other genes, has been consistently linked to elevated BMI in genome-wide association studies (23). A large-scale genome-wide study of childhood obesity further identified rs1059491 as a body fat-related variant (*P* = 2.57×10^-28^) and validated *SULT1A2* as an eQTL for obesity-relevant genes (24). Our data extend these genetic observations to the protein level, showing that circulating SULT1A2 is elevated in overweight states.

Nevertheless, we observed that SULT1A2 concentrations were highest in the overweight group rather than increasing progressively with obesity class. This non-monotonic distribution warrants a cautious biological interpretation. Several non-mutually exclusive explanations may account for this pattern. First, as a conventional anthropometric index, BMI cannot fully capture actual adiposity or metabolic burden, and the overweight category may encompass metabolically heterogeneous subgroups. Second, the association between adiposity and SULT1A2 levels may follow a threshold or bell-shaped response rather than a linear dose-response, possibly reflecting compensatory or counter-regulatory mechanisms. Third, unmeasured metabolic parameters, such as insulin sensitivity, inflammatory cytokines, or adipokine profiles, may serve as residual confounders underlying this unexpected finding. These possibilities remain speculative and highlight the exploratory nature of this observation.

Notably, baseline circulating SULT1A2 did not differ significantly between participants who achieved greater versus lesser weight loss in either sex. This discrepancy with the obesity-associated finding is likely due to the tissue-specific expression of SULT1A2, whereby serum levels cannot accurately mirror its local activity in key metabolic tissues such as adipose tissue, liver, or hypothalamic regions. Thus, the present data support a link between SULT1A2 and adiposity, but they do not provide evidence for its utility as a correlate of intervention-induced weight reduction.

### 4.2 Baseline SH2B1: from central physiology to peripheral exploratory candidate

SH2B1 is a well-characterized adaptor protein that modulates leptin and insulin signaling cascades and is indispensable for systemic energy homeostasis, appetite control, and adipose metabolism (17). Animal studies have demonstrated that deletion of SH2B1 in paraventricular hypothalamus neurons leads to energy imbalance, obesity, insulin resistance, and other metabolic disorders (25), with concordant reductions in hypothalamic SH2B1 mRNA and protein levels observed in obese mice (26). Moreover, SH2B1 expression in leptin-receptor-expressing neurons supports sympathetic nervous system function and protects against obesity and metabolic disturbances (27). In humans, chromosomal deletions encompassing *SH2B1* are associated with early-onset obesity and type 2 diabetes, supporting a protective role for SH2B1 against obesity (28, 29).

In contrast to this protective central paradigm, our study found that circulating SH2B1 levels were higher in overweight or obese participants than in their normal-weight counterparts. This seemingly paradoxical result may reflect a compensatory peripheral upregulation of circulating SH2B1 in response to obesity-related metabolic stress. Obesity is characterized by chronic leptin and insulin resistance, which could trigger a feedback increase in systemic SH2B1 production in an attempt to enhance leptin/insulin signaling and counteract metabolic dysfunction. Thus, elevated serum SH2B1 in obese individuals might represent a peripheral adaptive response, even in the setting of impaired central (e.g., hypothalamic) SH2B1 signaling.

However, several critical distinctions must be emphasized. Most previous studies have focused on tissue-specific (e.g., hypothalamic) SH2B1 expression or genetic variants, whereas our study measured circulating protein levels. Discrepancies between tissue and circulating concentrations have been documented for other adipokines and signaling proteins. For example, adipose tissue gene expression of SFRP2 and HILPDA does not correlate with their circulating levels, implying that local tissue changes may not translate into systemic blood concentrations (30), and cerebrospinal fluid versus plasma proteins concentrations are often poorly correlated (31). These precedents underscore that peripheral SH2B1 cannot be used as a direct readout of its central function activity.

Furthermore, the isoforms of SH2B1 detected in the peripheral circulation may differ from those predominantly expressed in neural tissues. Among the four alternatively spliced isoforms of SH2B1, SH2B1β and SH2B1γ are ubiquitously expressed, while SH2B1α and SH2B1δ are predominantly restricted to the brain. Murine studies have shown that knockout of brain-specific SH2B1α/δ isoforms confers resistance to obesity (16). Neuronal SH2B1β and SH2B1γ isoforms do not function as primary regulators of systemic energy homeostasis. Non-neuronal SH2B1β and SH2B1γ, together with neuronal SH2B1α and SH2B1δ, are sufficient for body weight maintenance; nevertheless, SH2B1β and SH2B1γ exert more prominent effects on weight reduction (32). Accordingly, modulation of SH2B1 splicing and isoform balance, alongside SH2B1α/δ suppression, has therefore been proposed as a promising therapeutic strategy, but our circulating measurements cannot distinguish these isoforms, further limiting mechanistic inference.

With respect to weight-loss outcomes, higher baseline circulating SH2B1 was observed exclusively in males who experienced greater weight loss, suggesting a sex-specific exploratory association. This sexual dimorphism is supported by animal studies that male SH2B1β/γ knockout mice exhibited mild increases in body weight, fat mass and food intake, whereas these alterations were absent in female mice (32). Deletion of brain-specific SH2B1α/δ also exerts sex-dependent metabolic effects, with males showing earlier and more substantial weight loss from 8 weeks of age, whereas females exhibit delayed and milder alterations until 20 weeks (16). Similarly, predictors of weight loss after bariatric surgery differed by sex, with insulin signaling indices being predictive in males and non-metabolic factors predominating in females (33). Collectively, these lines of evidence are consistent with the notion that SH2B1 may mediate male-specific weight regulation, primarily through insulin-leptin signaling pathways.

Genetic studies further support a role for SH2B1 variants in body-weight regulation and intervention responses (34). The SNP rs7359397 in SH2B1 has been reported to mediate divergent therapeutic responses in patients with non-alcoholic fatty liver disease following energy restriction (35), and the CpG-SNP rs7359397 is associated with longitudinal changes in body weight, BMI, and trunk fat mass in obese individuals on energy restriction regimens (36). Additionally, SH2B1 variants modulate not only BMI but also obstructive sleep apnea severity via leptin signaling, with wild-type alleles of rs4788102 and rs7359397 independently increasing risk apart from adiposity (37). These genetic observations lend biological plausibility to our exploratory protein-level findings but do not confirm a direct functional role for circulating SH2B1 in determining weight-loss magnitude.

### 4.3 Limitations

Several limitations should be acknowledged. First, the modest sample size precluded multivariable regression analyses simultaneously adjusting for multiple baseline covariates (body weight, BMI, visceral fat area, triglyceride levels, and other cardiometabolic variables). Consequently, we cannot exclude potential confounding of the observed association between SH2B1 and weight-loss response.

Second, unmeasured confounders, such as personal lifestyle, residential environment, educational attainment, and cognitive status, were not adjusted for, which may lead to overestimation of the observed effects. Third, despite using age- and sex-adjusted ANCOVA, the marked sex imbalance across weight groups (26.5% men in normal weight vs. 68.1% in obesity, *P* < 0.001) remains a concern. Given that sex may modulate SH2B1 function, residual sex-related confounding cannot be ruled out. Fourth, the use of a single≥5% relative weight-loss cutoff across varying baseline weights is a methodological limitation, as an identical proportional loss may have different physiological implications depending on initial body size, potentially biasing the grouping based on weight-loss magnitude and the observed SH2B1-weight loss association. Fifth, subgroup analyses were constrained by limited statistical power. *Post-hoc* power for the comparison between males with greater weight loss (n=10) versus those with lesser weight loss (n=7) was only 52.1% (Cohen’s d=1.06, α=0.05), well below the recommended 80% threshold, Accordingly, all reported *P*-values in subgroup analyses should be interpreted as descriptive and exploratory rather than confirmatory. These findings thus require validation in larger cohorts.

Sixth, the 4-week intervention period is relatively short. Early weight loss results from a mixture of fat loss, glycogen depletion, and fluid shifts, and the lack of separate body composition measurements complicates interpretation of the metabolic changes underlying our observations. Seventh, the 23.86% dropout rate represents another limitation. Although baseline profiles were balanced between completers and non-completers, unmeasured differences between these groups cannot be excluded, and the small sample (n=49) may obscure weak but potentially relevant biomarker effects, requiring validation in larger cohorts.

Eighth, all participants were recruited from a single center in Taizhou, China. Such single-center design may introduce selection bias and limit the representativeness of the study population, thereby limiting the generalizability and external validity of our findings. Finally, serum SH2B1 may not fully reflect central or tissue-specific SH2B1 activity, given the undefined linkage between peripheral and central compartments. This fundamental limitation precludes definitive mechanistic inference from our circulating biomarker data and reinforces the exploratory framing of our results.

## 5 Conclusions

In summary, circulating SULT1A2 and SH2B1 concentrations varied across adiposity phenotypes. Baseline serum SH2B1 was selectively associated with subsequent weight loss magnitude in males, specifically, higher baseline levels were observed in those who achieved greater weight reduction. However, this association did not remain significant after adjustment for age and BMI. Therefore, SH2B1 is an exploratory peripheral candidate that generates biologically plausible hypotheses regarding sex-specific energy-balance regulation. The observed associations require rigorous validation in larger, independent, and prospectively designed cohorts with standardized intervention protocols, alongside mechanistic studies capable of distinguishing isoform-specific and tissue-specific contributions.

## Data Availability

The raw data supporting the conclusions of this article will be made available by the authors, without undue reservation.
