# Supplementary material for: Serum SH2B1 and SULT1A2 in relation to adiposity and early weight-loss response: a hypothesis-generating study
Source: Front Endocrinol (Lausanne). 2026 Aug 6;17:1873732. doi: 10.3389/fendo.2026.1873732 (PMC13489798; doi:10.3389/fendo.2026.1873732)
Supplement: Supplementary file 1 [file Table3.docx]

| **Supplementary Table 3. Baseline anthropometric and cardiometabolic parameters** for females in the **greater and lesser** weight loss response to the diet intervention (n=18) | | | | | | | | |
| --- | --- | --- | --- | --- | --- | --- | --- | --- |
| Variables | Greater weight  loss group | Lesser weight  loss group | MD (High−Low) | MD 95%CI | *P* | *Cohen's d* | *Hedges’g* | *Hedges' g 95%CI* |
|  | n=14 | *n*=4 |  |  |  |  |  |  |
| Body weight (kg) | 72.0 ± 7.8 | 70.6 ± 7.1 | 1.38 | -7.85–10.61 | 0.755 | 0.18 | 0.171 | -0.891—1.229 |
| BMI (kg/m^2^) | 28.5 ± 2.9 | 28.2 ± 2.1 | 0.39 | -2.93–3.70 | 0.808 | 0.14 | 0.133 | -0.928—1.19 |
| SBP (mmHg) | 124 ± 18 | 131 ± 31 | -6.29 | -31.83–19.26 | 0.609 | -0.296 | -0.282 | -1.34—0.785 |
| DBP (mmHg) | 76 ± 14 | 79 ± 23 | -2.89 | -22.33–16.55 | 0.756 | -0.179 | -0.17 | -1.227—0.892 |
| Pulse (beat/min) | 77 ± 7 | 78 ± 4 | -0.46 | -6.79–5.86 | 0.873 | -0.068 | -0.064 | -1.122—0.995 |
| FPG (mmol/L) | 5.25 ± 1.3 | 4.81 ± 0.31 | 0.44 | -0.97–1.85 | 0.520 | 0.373 | 0.355 | -0.716—1.415 |
| TG (mmol/L) | 1.92 ± 1.42 | 1.04 ± 0.14 | 0.88 | -0.66–2.42 | 0.242 | 0.689 | 0.657 | -0.435—1.729 |
| TC (mmol/L) | 4.91 ± 1.01 | 5.62 ± 1.24 | -0.71 | -1.98–0.56 | 0.254 | -0.671 | -0.639 | -1.711—0.451 |
| LDL-C (mmol/L) | 2.49 ± 0.67 | 2.97 ± 0.96 | -0.48 | -1.36–0.40 | 0.26 | -0.662 | -0.63 | -1.701—0.459 |
| HDL-C (mmol/L) | 1.36 ± 0.14 | 1.62 ± 0.09 | -0.26 | -0.42 – (-0.10) | **0.003** | -1.98 | -1.886 | -3.105—-0.624 |
| Body Fat Mass | 30.1 ± 6.1 | 29.2 ± 4.4 | 0.89 | -6.06–7.84 | 0.790 | 0.154 | 0.146 | -0.915—1.203 |
| Percent Body Fat | 41.5 ± 4.9 | 41.1 ± 3.9 | 0.42 | -5.28–6.12 | 0.878 | 0.088 | 0.084 | -0.976—1.141 |
| Abdomen Circumference | 96.6 ± 8.0 | 96.9 ± 5.5 | -0.23 | -9.41–8.95 | 0.958 | -0.03 | -0.029 | -1.087—1.03 |
| Waist Hip Ratio | 0.96 ± 0.05 | 0.98 ± 0.03 | -0.02 | -0.07–0.04 | 0.460 | -0.429 | -0.409 | -1.47—0.665 |
| Visceral Fat Area | 155.4 ± 34.9 | 155.1 ± 26.6 | 0.29 | -39.99–40.58 | 0.988 | 0.009 | 0.008 | -1.05—1.066 |
| SULT1A2 (ng/mL) | 16.35 ± 6.00 | 17.65 ± 1.91 | -1.30 | -7.87 – 5.27 | 0.498 | -0.237 | -0.226 | -1.283—0.839 |
| SH2B1 (ng/mL) | 1520.7 ± 881.2 | 1501.5 ± 216.3 | 19.22 | -942.01 – 980.46 | 0.942 | 0.024 | 0.023 | -1.036—1.081 |

Participants were classified as greater weight loss group if they achieved ≥5% reduction in initial body weight, and lesser weight loss group if weight loss was <5%.

Ref: Aller EEJG, Mariman ECM, Bouwman FG, van Baak MA. Genetic Predictors of ≥5% Weight Loss by Multidisciplinary Advice to Severely Obese Subjects. J Nutrigenet Nutrigenomics. 2017;10(1-2):32-42. doi: 10.1159/000469662.

MD, mean difference; CI, confidence interval.

Bold values represent statistically significant results with *P* < 0.05.
