# Supplementary material for: Serum SH2B1 and SULT1A2 in relation to adiposity and early weight-loss response: a hypothesis-generating study
Source: Front Endocrinol (Lausanne). 2026 Aug 6;17:1873732. doi: 10.3389/fendo.2026.1873732 (PMC13489798; doi:10.3389/fendo.2026.1873732)
Supplement: Supplementary file 2 [file Table4.docx]

| **Supplementary Table 4. Baseline anthropometric and cardiometabolic parameters** for males **in the greater and lesser weight loss response to the diet intervention (*n*=17)** | | | | | | | | |
| --- | --- | --- | --- | --- | --- | --- | --- | --- |
| Variables | Greater weight  loss group | Lesser weight  loss group |  |  |  |  |  |  |
|  | n=10 | n=7 | *MD (High−Low)* | *MD 95%CI* | *P* | *Cohen's d* | *Hedges’g* | *Hedges' g 95%CI* |
| Body weight (kg) | 89.3 ± 9.0 | 92.2 ± 13.1 | -2.91 | -14.30–8.49 | 0.594 | -0.27 | -0.25 | -1.17—0.67 |
| BMI (kg/m^2^) | 30.5 ± 3.8 | 31.2 ± 3.2 | -0.74 | -4.46–2.98 | 0.678 | -0.21 | -0.20 | -1.11—0.72 |
| SBP (mmHg) | 140 ± 22 | 136 ± 21 | 4.11 | -18.51–26.74 | 0.704 | 0.19 | 0.18 | -0.74—1.10 |
| DBP (mmHg) | 88 ± 22 | 82 ± 16 | 6.09 | -14.46–26.64 | 0.537 | 0.31 | 0.30 | -0.63—1.21 |
| Pulse (beat/min) | 85 ± 13 | 83 ± 11 | 1.37 | -11.63–14.38 | 0.825 | 0.11 | 0.11 | -0.81—1.02 |
| FPG (mmol/L) | 5.81 ± 1.81 | 5.42 ± 1.02 | 0.39 | -1.23–2.02 | 0.613 | 0.26 | 0.24 | -0.68—1.16 |
| TG (mmol/L) | 3.23 ± 3.78 | 1.78 ± 0.83 | 1.45 | -1.67–4.58 | 0.336 | 0.49 | 0.46 | -0.47—1.39 |
| TC (mmol/L) | 5.03 ± 0.90 | 4.92 ± 0.82 | 0.12 | -0.80–1.03 | 0.790 | 0.13 | 0.13 | -0.79—1.04 |
| LDL-C (mmol/L) | 2.41 ± 0.62 | 2.69 ± 0.58 | -0.27 | -0.91–0.36 | 0.370 | -0.46 | -0.43 | -1.35—0.50 |
| HDL-C (mmol/L) | 1.13 ± 0.19 | 1.17 ± 0.27 | -0.03 | -0.27–0.20 | 0.760 | -0.15 | -0.15 | -1.06—0.78 |
| Body Fat Mass | 27.8 ± 7.8 | 31.8 ± 5.7 | -4.09 | -11.50–3.32 | 0.258 | -0.58 | -0.55 | -1.48—0.40 |
| Percent Body Fat | 30.7 ± 6.2 | 34.4 ± 3.9 | -3.73 | -9.42–1.95 | 0.182 | -0.69 | -0.65 | -1.59—0.30 |
| Abdomen Circumference | 103.2 ± 10.9 | 105.2 ± 10.5 | -1.93 | -13.19–9.34 | 0.720 | -0.18 | -0.17 | -1.09—0.75 |
| Waist Hip Ratio | 0.96 ± 0.06 | 0.96 ± 0.06 | 0.00 | -0.07–0.06 | 0.974 | -0.02 | -0.02 | -0.93—0.9 |
| Visceral Fat Area | 124.0 ± 42.3 | 142.5 ± 28.7 | -18.47 | -57.81–20.87 | 0.333 | -0.49 | -0.47 | -1.39—0.47 |
| SULT1A2 (ng/mL) | 22.42 ± 5.03 | 24.75 ± 5.37 | -2.33 | -7.76–3.10 | 0.375 | -0.45 | -0.43 | -1.35—0.51 |
| SH2B1 (ng/mL) | 2477.1 ± 1332.2 | 1298.4 ± 649.6 | 1178.70 | 12.05–2345.35 | **0.030** | 1.06 | 1.01 | 0.01—1.98 |

Participants were classified as greater weight loss group if they achieved ≥5% reduction in initial body weight, and lesser weight loss group if weight loss was <5%.

Ref: Aller EEJG, Mariman ECM, Bouwman FG, van Baak MA. Genetic Predictors of ≥5% Weight Loss by Multidisciplinary Advice to Severely Obese Subjects. J Nutrigenet Nutrigenomics. 2017;10(1-2):32-42. doi: 10.1159/000469662.

MD, mean difference; CI, confidence interval.

Bold values represent statistically significant results with *P* < 0.05.
