# Supplementary material for: Serum SH2B1 and SULT1A2 in relation to adiposity and early weight-loss response: a hypothesis-generating study
Source: Front Endocrinol (Lausanne). 2026 Aug 6;17:1873732. doi: 10.3389/fendo.2026.1873732 (PMC13489798; doi:10.3389/fendo.2026.1873732)
Supplement: Supplementary file 3 [file Table1.doc]

**Supplementary** **Table 1. Baseline anthropometric and cardiometabolic parameters for female in the greater and lesser weight loss response to the diet intervention (n=32)**

| Variables | Greater weight loss group | Lesser weight loss group | *P* | M ( (High−Low)) | 95%CI |
| --- | --- | --- | --- | --- | --- |
|  | n=20 | n=12 |  |  |  |
| Body weight (kg) | 67.2 (63.0-72.8) | 63.0 (61.4-69.2) | 0.272 | 3.4 | -1.6–8.9 |
| BMI (kg/m^2^) | 26.7 (24.4-29.6) | 24.5 (23.5-27.4) | 0.083 | 1.85 | -0.24–4.16 |
| SBP (mmHg) | 121 (112-134.5) | 118 (107-125) | 0.326 | 5 | -6–16 |
| DBP (mmHg) | 72 (65-79) | 72 (66-80) | 0.893 | 1 | -7–13 |
| Pulse (beat/min) | 78 (71-85) | 79 (73-84) | 0.833 | -1 | -7–6 |
| FPG (mmol/L) | 4.98 (4.50-5.44) | 4.99 (4.72-5.20) | 0.833 | -0.03 | -0.41–0.37 |
| TG (mmol/L) | 1.33 (1.03-1.91) | 1.01 (0.76-1.21) | **0.029** | 0.37 | 0.04–0.83 |
| TC (mmol/L) | 4.74 (4.09-5.33) | 4.56 (4.03-5.66) | 0.924 | 0.03 | -0.78–0.72 |
| LDL-C (mmol/L) | 2.28 (2.02-2.89) | 2.25 (1.99-3.23) | 0.999 | 0 | -0.58–0.45 |
| HDL-C (mmol/L) | 1.35 (1.25-1.51) | 1.51 (1.20-1.71) | 0.209 | -0.14 | -0.33–0.06 |
| Body Fat Mass | 27.7 (22.0-30.0) | 23.4 (21.9-27.5) | 0.239 | 3.25 | -1.4–6.5 |
| Percent Body Fat | 40.0 (34.7-43.2) | 36.9 (33.5-39.9) | 0.224 | 2.65 | -1.4–6.4 |
| Abdomen Circumference | 92.6 (88.6-98.2) | 88.9 (86.3-96.7) | 0.182 | 3.6 | -2.1–8.6 |
| Waist Hip Ratio | 0.95 (0.91-0.98) | 0.93 (0.91-0.97) | 0.578 | 0.01 | -0.02–0.04 |
| Visceral Fat Area | 145.8 (108.3-157.1) | 116.4 (105.8-139.2) | 0.224 | 18.2 | -11.4–41.8 |
| SULT1A2 (ng/mL) | 15.86 (12.33-19.46) | 16.59 (13.25-19.06) | 0.803 | -0.496 | -4.136–3.096 |
| SH2B1 (ng/mL) | 1160.13 (873.58-1448.78) | 1055.66 (759.01-1568.83) | 0.632 | 84.771 | -347.002–474.22 |

Participants were classified as greater weight loss group if they achieved ≥5% reduction in initial body weight, and lesser weight loss group if weight loss was <5%.

Ref: Aller EEJG, Mariman ECM, Bouwman FG, van Baak MA. Genetic Predictors of ≥5% Weight Loss by Multidisciplinary Advice to Severely Obese Subjects. J Nutrigenet Nutrigenomics. 2017;10(1-2):32-42. doi: 10.1159/000469662.

Data were expressed as median and interquartile range. M, median; CI, confidence interval. *P* values are obtained based on the Mann-Whitney *U* test.

Bold values represent statistically significant results with *P* < 0.05.
