# Supplementary material for: Serum SH2B1 and SULT1A2 in relation to adiposity and early weight-loss response: a hypothesis-generating study
Source: Front Endocrinol (Lausanne). 2026 Aug 6;17:1873732. doi: 10.3389/fendo.2026.1873732 (PMC13489798; doi:10.3389/fendo.2026.1873732)
Supplement: Supplementary file 4 [file Table2.doc]

**Supplementary Table 2. Baseline anthropometric and cardiometabolic parameters for male in the greater and lesser weight loss response to the diet intervention (n=17)**

| Variables | Greater weight loss group | Lesser weight loss group | *P* | M | 95%CI |
| --- | --- | --- | --- | --- | --- |
|  | *n*=10 | *n*=7 |  |  |  |
| Body weight (kg) | 86.7 (81.9-98.1) | 97.3 (77.3-102) | 0.601 | -6.7 | -16.4–9.8 |
| BMI (kg/m^2^) | 29.8 (26.6-34.4) | 31.9 (27.4-33.1) | 0.669 | -1.03 | -5.45–3.09 |
| SBP (mmHg) | 135 (128-148) | 143 (111-149) | 0.999 | -0.5 | -19–27 |
| DBP (mmHg) | 78 (76-100) | 82 (63-90) | 0.962 | -1 | -14–27 |
| Pulse (beat/min) | 91 (79-94) | 86 (71-95) | 0.887 | 1.5 | -9–19 |
| FPG (mmol/L) | 5.06 (4.67-6.25) | 4.98 (4.68-6.57) | 0.887 | 0.03 | -0.59–1.33 |
| TG (mmol/L) | 1.67 (1.3-3.66) | 1.59 (1.29-1.6) | 0.74 | 0.135 | -0.3–2.09 |
| TC (mmol/L) | 4.78 (4.41-5.69) | 5.35 (4.08-5.67) | 0.887 | 0.09 | -1.03–1.22 |
| LDL-C (mmol/L) | 2.42 (1.88-3.1) | 2.7 (2.16-3.17) | 0.475 | -0.29 | -0.89–0.41 |
| HDL-C (mmol/L) | 1.17 (1.02-1.26) | 1.14 (0.98-1.17) | 0.475 | 0.04 | -0.28–0.2 |
| Body Fat Mass | 24.6 (21.3-34.1) | 32.5 (28.3-36.7) | 0.193 | -5.6 | -12.6–3.5 |
| Percent Body Fat | 28.7 (25.6-36.7) | 34.9 (31.8-37.2) | 0.161 | -4 | -10.5–3.5 |
| Abdomen Circumference | 102.8 (91.6-112.9) | 107.6 (92.3-111.4) | 0.813 | -1.5 | -15.8–11 |
| Waist Hip Ratio | 0.96 (0.89-1.02) | 0.95 (0.92-1.02) | 0.999 | 0 | -0.07–0.07 |
| Visceral Fat Area | 105 (91.1-159.4) | 147.1 (135.9-156.5) | 0.475 | -33.55 | -58.9–17.1 |
| SULT1A2 (ng/mL) | 22.55 (17.91-26.72) | 25.77 (18.2-28.43) | 0.475 | -1.862 | -8.755–2.636 |
| SH2B1 (ng/mL) | 2597.65 (1162.8-3741.16) | 1159.82 (992.48-1435.52) | 0.088 | 1281.298 | -139.238–2581.609 |

Participants were classified as greater weight loss group if they achieved ≥5% reduction in initial body weight, and lesser weight loss group if weight loss was <5%.

Ref: Aller EEJG, Mariman ECM, Bouwman FG, van Baak MA. Genetic Predictors of ≥5% Weight Loss by Multidisciplinary Advice to Severely Obese Subjects. J Nutrigenet Nutrigenomics. 2017;10(1-2):32-42. doi: 10.1159/000469662.

Data were expressed as median and interquartile range. M, median; CI, confidence interval. *P* values are obtained based on the Mann-Whitney *U* test.
