# Supplementary material for: Serum SH2B1 and SULT1A2 in relation to adiposity and early weight-loss response: a hypothesis-generating study
Source: Front Endocrinol (Lausanne). 2026 Aug 6;17:1873732. doi: 10.3389/fendo.2026.1873732 (PMC13489798; doi:10.3389/fendo.2026.1873732)
Supplement: Supplementary file 5 [file Table5.doc]

**Supplementary Table 5. Associations of baseline serum SH2B1 and SULT1A2 protein levels with weight loss response to the diet intervention in logistic regression models**

|  |  | Model 1 (Unadjust) | | |  | Model 2 (adjusted for age and BMI at baseline) | | |
| --- | --- | --- | --- | --- | --- | --- | --- | --- |
| Variables | Sex | *OR* | 95% *CI* | *P* |  | *OR* | 95% *CI* | *P* |
| SH2B1 |  |  |  |  |  |  |  |  |
|  | All (n=49) | 1.001 | 1.000-1.001 | 0.089 |  | 1.001 | 1.000-1.002 | 0.106 |
|  | Men (n=17) | 1.001 | 1.000-1.002 | 0.082 |  | 1.001 | 1.000-1.003 | 0.16 |
|  | Women (n=32) | 1.000 | 0.999-1.002 | 0.464 |  | 1.000 | 0.999-1.001 | 0.811 |
| SULT1A2 |  |  |  |  |  |  |  |  |
|  | All (n=49) | 0.961 | 0.873-1.057 | 0.413 |  | 0.978 | 0.866-1.103 | 0.715 |
|  | Men (n=17) | 0.908 | 0.740-1.113 | 0.351 |  | 0.958 | 0.749-1.225 | 0.733 |
|  | Women (n=32) | 0.976 | 0.845-1.127 | 0.738 |  | 0.969 | 0.833-1.126 | 0.678 |
